# Genotype and clinical phenotype of children with Marfan syndrome in Southeastern Anatolia

**DOI:** 10.1007/s00431-024-05579-3

**Published:** 2024-05-03

**Authors:** Murat Karaoglan, Gulper Nacarkahya, Emel Hatun Aytac, Mehmet Keskin

**Affiliations:** 1 Faculty of Medicine, Department of Pediatric Endocrinology, Gaziantep University, Gaziantep, Turkey; 2 Department of Molecular Biology, Gaziantep University Faculty of Medicine, Gaziantep, Turkey

**Keywords:** Marfan syndrome, Aortic dilatation, Ectopia lentis, *FBN1*

## Abstract

The cardinal phenotypic hallmarks of Marfan syndrome (MFS) include cardiac, ocular, and skeletal abnormalities. Since the clinical phenotype of MFS is highly heterogeneous, with certain symptoms appearing as children age, the diagnostic process and establishing a genotype-phenotype association in childhood MFS can be challenging. The lack of sufficient childhood studies also makes it difficult to interpret the subject. This study aims to evaluate the relationship between clinical symptoms used as diagnostic criteria and *FBN1* variations in children with MFS. This study investigated the relationships between genotypes and phenotypes in 131 children suspected of having Marfan syndrome (MFS). Diagnosis of MFS was made according to the revised Ghent nosology. *FBN1* variants were categorized based on exon regions, type of variant, and pathogenicity classes. These *FBN1* variants were then correlated with the clinical manifestations including cardiovascular, ocular, facial, and skeletal abnormalities. Out of the children, 43 were diagnosed with MFS. *FBN1* variant was identified in 32 (74.4%) of the MFS children. MFS diagnosis could not be made in five (15.6%) *FBN1* variant-positive children. The most common cardinal finding is cardiac anomalies *n* = 38 (88.3%). The most common *FBN1* pathogenic variant was c.1786 T > C/p.Cys596Arg *n* = 4 (12.5%). The distribution of pathogenic variants was as follows: 29 (90.6%) missense, 2 (6.3%) frameshift, and 1 (3.1%) nonsense. The numbers of AD and EL of the variant-positive children were 16 (50%) and 14 (43.7%), respectively. Ocular abnormalities were more common in children with *FBN1-*positive MFS (*p* = 0.009). There was no difference in the number of cardiac abnormalities between *FBN1*-positive and *FBN1*-negative MFS patients (*p* = 0.139).

*Conclusion*: This study examines the relationship between *FBN1* variants and clinical features used as diagnostic criteria in MFS children. The findings emphasize the importance of long-term monitoring of heterogeneous clinical phenotypes and bioinformatic reanalysis in determining the genotype-phenotype relationship in children, as MFS symptoms can vary with age.

**Table:** 

**What is Known:** *• Marfan syndrome has highly variable phenotypic heterogeneity.* *• The genotype-phenotype relationship in childhood Marfan syndrome is not clear enough due to the variation in the time of onset of the findings.*	
**What is New:** *• This article provides regional data for the field of research on genotype-phenotype relationships in childhood Marfan syndrome.* *• Long-term follow-up of clinical findings and bioinformatics reanalysis is an important requirement for a well-established genotype-phenotype relationship in childhood Marfan syndrome.*

**What is Known:**

*• Marfan syndrome has highly variable phenotypic heterogeneity.*

*• The genotype-phenotype relationship in childhood Marfan syndrome is not clear enough due to the variation in the time of onset of the findings.*

**What is New:**

*• This article provides regional data for the field of research on genotype-phenotype relationships in childhood Marfan syndrome.*

*• Long-term follow-up of clinical findings and bioinformatics reanalysis is an important requirement for a well-established genotype-phenotype relationship in childhood Marfan syndrome.*

## Introduction

Marfan syndrome (MFS) is a type 1 fibrillinopathy, a connective tissue disease caused by *FBN1* pathogenic variants [1, 2]. The main clinical findings involve the cardiovascular (CVS), ocular, and skeletal systems. Additionally, patients may exhibit cutaneous, respiratory, integumental, and nervous system findings [3]. MFS is an autosomal dominant disease, with approximately 25% of cases resulting from de novo variants [4, 5].

One of the most common manifestations is cardiovascular findings, which occur in 60–80% of cases and are the leading cause of morbidity and premature death [6]. The typical ocular finding is ectopia lentis (EL). However, the most distinct phenotype in MFS is characterized by skeletal system findings [7]. The diagnosis of MFS is based on the revised Ghent nosology [8]. The criteria for diagnosis included family history, positivity for *FBN1* variants, aortic dilatation, presence of ectopic lens, and systemic findings.

Research on the genotype-phenotype relationship in Marfan syndrome plays a crucial role in establishing prognostic criteria for other potential systemic findings that can result in disability, particularly cardiac anomalies that can lead to premature death [9]. Although conflicting results have been reported in previous studies, it has been found that onset under 1 year of age, aortic involvement, diaphragmatic herniation, and neonatal exon region variants are associated with early death or a severe clinical course [10].

Establishing a genotype-phenotype relationship in children with MFS has several important limitations, and there are not enough studies on the subject [2]. Firstly, clinical manifestations are highly heterogeneous, with the clinical phenotype displaying highly variable expression. Phenotypic variability and penetrance can vary even among individuals within the same family [9]. Secondly, since the characteristic findings of the disease develop over time, diagnosing and predicting prognosis in pediatric patients can be challenging [11]. The timing of symptom onset and the specific distribution of manifestations in different tissues contribute to phenotypic variability [12]. Previous studies have reported that aortic anomalies, ectopic lens, and specific skeletal findings differ between individuals under and above the age of 18 [13, 14]. Thirdly, other disorders with symptoms similar to MFS, such as mitral valve prolapse syndrome or Shprintzen-Goldberg syndrome, can complicate the genotype-phenotype correlation due to fibrillinopathies caused by TGFβRII variants [15, 16]. Inconsistencies between the revised Ghent criteria developed for diagnostic purposes and *FBN1* variant positivity can sometimes make it difficult to establish both a diagnosis and genotype-phenotype correlation [17].

The aim of this study is to investigate the genotype and phenotype characteristics used as diagnostic criteria for Marfan syndrome in Turkish children.

## Materials and methods

This retrospective study was conducted at a single center and involved 131 children who underwent *FBN1* screening due to suspicion of Marfan syndrome between 2016 and 2021.

**Fig. 1:**
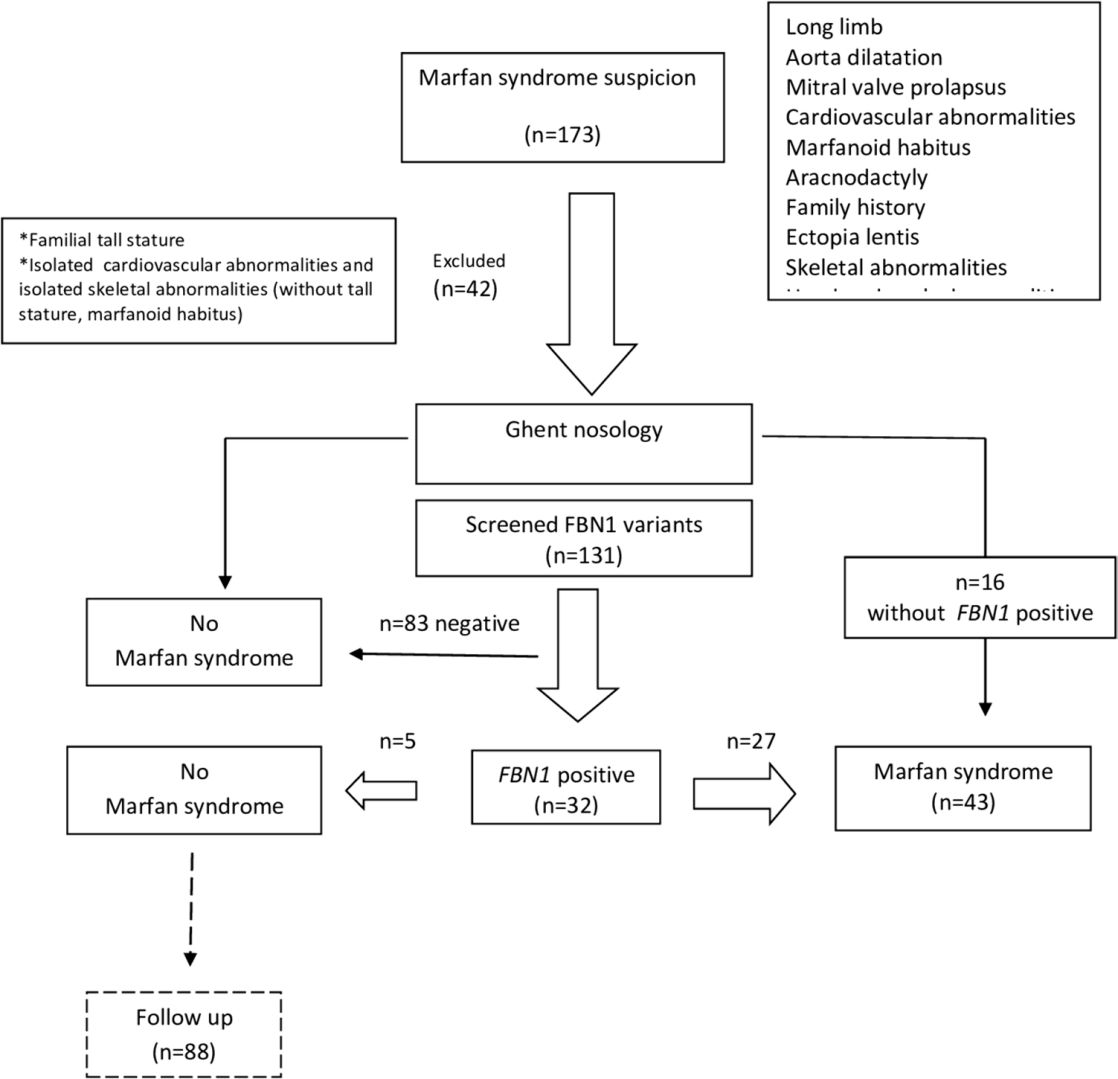
Flow chart of the selection and diagnosis process for cases screened with suspicion of Marfan syndrome

One hundred seventy-three patients were referred from orthopedics, ophthalmology, cardiology, and pediatrics outpatient clinics with suspicion of MFS (Fig. 1). Clinical findings suggestive of MFS included tall stature (height >  +2.5 SDS), family history, arachnodactyly, lens subluxation, and anomalies of the chest, face, and extremities. *FBN1* screening was not performed in 42 patients with another specific diagnosis: familial tall stature (*n* = 34), isolated cardiovascular findings (*n* = 4), and skeletal system findings (*n* = 4). *FBN1* screening was performed to 131 patients who presented with at least one MFS-related finding [18]. These findings included a family history, clinical signs, and symptoms suggestive of Marfan syndrome, such as a Marfanoid appearance (long limbs, long face, thumb sign, wrist sign), aortic dilatation, other specific cardiovascular findings, skeletal system abnormalities, or ectopia lentis. In addition, a total of 131 patients underwent *FBN1* molecular analysis. *FBN1* screening was conducted on children who could not be diagnosed with MFS according to the revised Ghent criteria but were strongly suspected of having MFS. These children were mostly those for whom specific findings were expected to develop with age. Additionally, *FBN1* screening was scheduled for children whose height exceeded their midparental height. Several cases were referred from various departments (ophthalmology, orthopedics, cardiology, etc.) with an *FBN1* analysis report. The researchers collected clinical signs, symptoms, and genotype characteristics. Out of the 43 patients diagnosed with MFS according to the Ghent nosology criteria, 16 were found to have the *FBN1* variant. The diagnosis of MFS was excluded in 88 cases, with the *FBN1* variant detected in 5 participants.

To describe clinical signs and symptoms of Marfan syndrome, annotations were sourced from the Human Phenotype Ontology, a standardized dictionary of phenotypic abnormalities in human diseases. These annotations covered a range of phenotypic abnormalities including growth (disproportionate tall stature, slender build), head and neck (long and narrow face, dental crowding, high narrow palate, downslanted palpebral fissures, open bite), eye (ectopia lentis, lens subluxation, lens luxation, glaucoma, retinal detachment, hypoplasia of the iris, increased axial length of the globe, flat cornea visual impairment, myopia), cardiovascular (ascending tubular aorta aneurysm, ascending aortic dissection, arterial dissection, mitral valve prolapse, tricuspid regurgitation, mitral regurgitation, bicuspid aorta, congestive heart failure), skeletal system (pectus carinatum, pectus excavatum, arachnodactyly, joint hypermobility, scoliosis, protrusio acetabuli, abnormal zygomatic bone morphology, dolichocephaly, retrognathia, micrognathia, kyphosis, limited elbow movement, osteoporosis, mitral valve calcification), and nervous system (dural ectasia). Marfanoid habitus was defined as a set of clinical signs and symptoms, with the most common features being disproportionate tall stature, long arms and legs, arachnodactyly, and joint hypermobility.

The researchers gathered data on family history, cardiac and ocular findings, and skeletal findings. They also documented measurements of height, weight, and body mass index. Aortic diameter *Z*-scores were extracted from echocardiography records. Aortic diameters were calculated using an automated software created by Cantinotti et al. [19]. The program captured measurements such as aortic valve annulus (mm), sinus of Valsalva (mm), sino-tubular junction (mm), and ascending aorta (mm) from the echocardiography results. The revised Ghent nosology was utilized to categorize phenotypes [18]. Systemic findings were employed as diagnostic criteria for cases with a score of ≥ 7. Marfan syndrome (MFS) was diagnosed based on the outlined criteria in the Ghent nosology.

### Molecular analysis

The NGS (next-generation sequencing) analysis method was utilized to analyze the *FBN1* gene. Primers covering the specified exons of the gene were employed. DNA was extracted from blood samples collected in EDTA tubes. The DNA concentration was measured using a Qubit 3.0 device (ThermoScientific, USA) and adjusted to a final concentration of 2 nanograms per microliter. Initial PCR steps (library preparation) were conducted with the obtained DNA samples and primer pairs specifically designed for sequencing the *FBN1* gene, not for complete panel sequencing. The library fragments acted as templates for synthesizing new DNA fragments. Following the library preparation phase, the manufacturer’s kit was utilized for the emulsion PCR steps, which were carried out on the Illumina MiSeq device (USA) according to their protocols. The digital recording of the introduction of nucleotides into the growing DNA helix took place. The sample mixtures were processed using the relevant kits on the Illumina MiSeq device and loaded onto the appropriate chips to initiate the sequencing stage.

### Variant classification

*FBN1* variants were identified as exon regions that exhibited a strong genotype-phenotype correlation based on previous data. Consequently, the exon regions were categorized into three groups: N-terminus (exon 1–21), middle region (exon 22–42), and C-terminus (exon 43–65). The relationship between genotypes in these regions and the manifestations in the patients was assessed.

The pathogenicity classification of variants was determined in accordance with the 2015 American College of Medical Genetics (ACMG) standards and guidelines [20]: Class 1 (benign), Class 2 (likely benign), Class 3 VUS (variant of unknown significance), Class 4 (likely pathogenic), and Class 5 (pathogenic). Specific guidelines for *FBN1* were utilized to determine variant pathogenicity based on ACMG [21]. The pathogenicity of the variants was compared to the phenotypic abnormalities.

Bioinformatics analyses were conducted using various databases (Encode, ClinVar, OMIM, COSMIC, PubMed, HGMD, PGMD, Ensemble, Mutation Taster, Varsome). The variants’ domains were identified using Prosit (http://www.expasy.org/prosite/) and Smart (https://www.smartgene.com/). The MutationTaster analysis program was employed for in silico analysis.

The cases were clinically divided into two groups based on *FBN1* positivity and cardiac findings: *FBN1* positive and negative groups, and cardiac findings positive and negative groups.

### Statistics

Quantitative variables were defined as mean ± standard deviation, while qualitative variables were expressed as percentages. Group comparisons were conducted using the chi-square (*χ*^2^) test, with the Fisher test used if the chi-square test was unsuccessful. The distribution was tested using the Kolmogorov-Smirnov test. The Student *t* test was used for normally distributed variables, and the Mann–Whitney *U* test was used for non-normally distributed variables. Statistical analyses were conducted using SPSS v21 (IBM Corporation, New York, USA).

## Results

Out of the 131 participants screened for *FBN1*, 72 (55%) were male. Table 1 displays the anthropometric and clinical characteristics of the participants (*n* = 131) who were screened for *FBN1*. It was found that CVS, ocular, and facial findings were significantly higher in the *FBN1* positive group (*p* < 0.001, *p* < 0.001, and *p* = 0.007), while no significant difference was observed between the groups in terms of the frequency of skeletal findings (*p* = 0.134). The most common manifestation was CVS findings (*n* = 61) (48%). Additional CVS findings can be found in Table 1.

**Table 1:** Comparison of clinical features of *FBN1* variant–positive and –negative cases

	***FBN1* ( +)** **(*n* = 32)**	***FBN1* ( −) (*n* = 99)**	***p***
**Boy/girl**	18/14	54/45	0.730
**Age at diagnosis**	11.59 ± 7.67	12.87 ± 5.66	0.279
**Height**	147.87 ± 10.60	160.63 ± 30.24	0.097
**Height SDS**	2.07 ± 0.20	1.81 ± 0.30	0.467
**Weight**	42.10 ± 48.08	52.40 ± 51.61	0.154
**Weight SDS**	0.3 ± 1.2	0.39 ± 2.13	0.730
**Family history**	4	4	0.006
**Signs/symptoms**			
**Cardiovascular abnormalities**	27	34	< 0.001^a^
**Aortic root dilatation**	16	12	< 0.001^a^
**Bicuspid aortic valve**	3	3	0.135
**Mitral valve prolapse**	20	16	< 0.001^a^
**Mitral regurgitation**	18	18	< 0.001^a^
**Tricuspid valve prolapse**	7	3	< 0.001^a^
**Tricuspid regurgitation**	7	9	0.548
**Aortic aneurysm**	1	0	0.244
**Aortic dissection**	1	1	0.430
**Ocular abnormalities**	14	4	< 0.001^a^
**Ectopia lentis**	14	4	< 0.001^a^
**Myopia**	2	4	0.270
**Skeletal abnormalities**	23	83	0.134
**Long limb**	22	47	0.036^a^
**Thumb sign**	20	31	0.016^a^
**Wrist sign**	16	17	0.002^a^
**Pectus deformities**	1	11	0.161
**Scoliosis**	0	9	0.112
**Hindfoot**	7	2	0.001^a^
**Joint laxity**	2	6	0.968
**Facial abnormalities**	14	10	0.007^a^
**Long and narrow face**	13	10	< 0.001^a^
**High arched palate**	4	3	0.038^a^
**Malar hypoplasia**	7	4	0.001^a^
**Micro/retrognathia**	8	2	< 0.001^a^

The Student *t* test (two-sided), chi-square, and Fischer’s exact test were used *p* < 0.05 is significant

^a^Abnormalities were more common in the FBN1 positive group

**Fig. 2:**
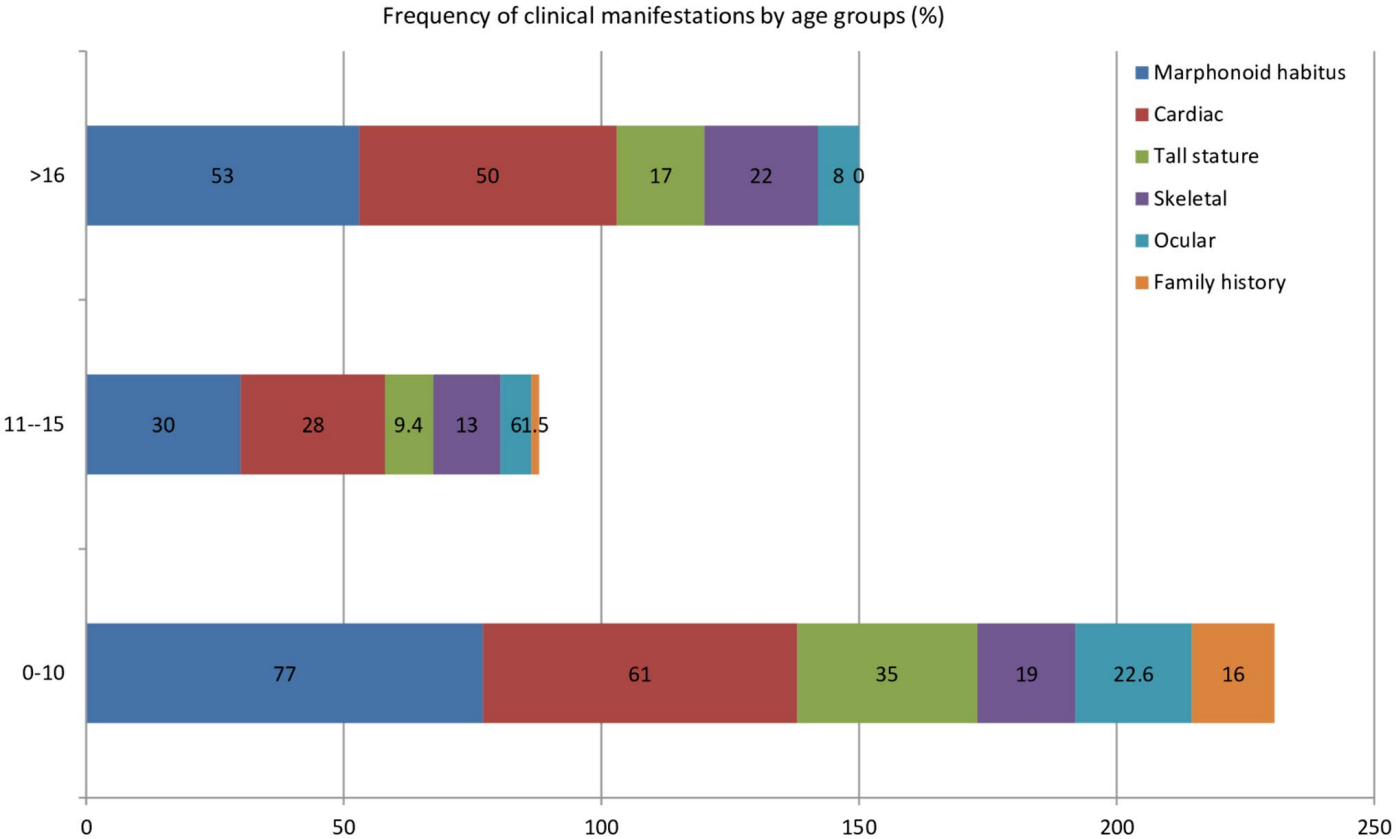
Percentage distribution of clinical manifestations by age groups

The mean age at diagnosis for cases with AD and EL was as follows: 12.5 ± 5.43 and 12.96 ± 4.79, respectively. Figure 2 shows the percentage distribution of clinical manifestations by age groups.

Tables 2 and 3 provide clinical phenotypes associated with the modified Ghent criteria. Out of all the participants, 43 children (32.82%) were diagnosed with Marfan syndrome (MFS). The *FBN1* variant was detected in 32 patients (74.4%) diagnosed with MFS in children. Out of the 32 *FBN1* variants, 27 (63%) were diagnosed with MFS, while the remaining 5 were still being followed up for a possible diagnosis of MFS. Among the patients with manifestations related to MFS, 5 patients were *FBN1* positive (3.8%), while 83 (63.35%) were *FBN1* negative according to the Ghent criteria.

**Table 2:** Patients diagnosed with Marfan syndrome based on the revised Ghent criteria

Criteria 1	Criteria 2	*n*	Explanation
Aorta diameter^a^	Ectopia lentis	2	No FBN1variant
Aorta diameter^a^	*FBN1* variants	27	All have ectopia lentis
Aorta diameter ^a^	Systemic score > 7	13	No FBN1variant
Family history	Systemic score > 7	1	No FBN1variant
Total		43	Marfan syndrome

^a^*Z*-score ≥ 3 in individuals < 20 years

**Table 3:** Clinical conditions related to the Ghent criteria

Criteria 1	Criteria 2	*n*	Explanation
Family history	Systemic score < 5	2	FBN1 negative
MVP, AD *Z*-score < 2	Systemic score ≥ 5, skeletal findng, *FBN1* ( −)	18	MVPS
*FBN1*(+)	Systemic score < 5	5	Unidentified
Ectopia lentis Systemic score < 7	FBN1 (−) FBN1 unrelated cardiac finding	2	Ectopia lentis syndrome
Systemic score < 5	Marfanoid habitus accompanying tall stature	61	Screening for Marfan syndrome suspicion
Total		88	

*FBN1* positive but no Marfan syndrome diagnosis (*n* = 5)

*FBN1* negative plus Marfan syndrome-related findings (*n* = 83)

*FBN1* negative but Marfan syndrome diagnosis (*n* = 16)

*AD* aorta diameter, *MVPS* mitral valve prolapse syndrome

There were 16 cases (12.21%) diagnosed with MFS without an *FBN1* variant (Table 4). Table 3 displays the anthropometric and clinical characteristics of patients diagnosed with MFS (*n* = 43) in relation to *FBN1* positivity. While there were no differences between the *FBN1* positive and negative groups in terms of CVS, skeletal, and facial findings (*p* = 1, *p* = 0.832, and *p* = 0.188), ocular findings were more frequently observed in the *FBN1* positive group (*p* = 0.009). Other findings are detailed in Table 4.

**Table 4:** Distribution of phenotypic abnormalities according to FBN1 pathogenic variant status in patients diagnosed with Marfan syndrome based on revised the Ghent nosology

	FBN1 positive (*n* = 27)/mean ± SD	FBN1negative (*n* = 16)/mean ± SD	*p*
**Male/female**	12/15	10/6	0.310
**Age (years)**	11.20 ± 5.42	13.18 ± 4.30	0.134
**Height SDS**	1.88 ± 1.14	1.19 ± 1.99	0.215
**Family history**	4	2	0.345
**Cardiovascular abnormalities**	22	16	0.139
**Aortic root dilatation**	16	12	0.295
**Mitral valve prolapse**	20	6	0.006^a^
**Mitral regurgitation**	18	5	0.024^a^
**Tricuspid regurgitation**	7	4	0.946
**Aortic bicuspid valve**	3	1	0,358
**Ascending aortic aneurysm**	1	0	0.549
**Aortic dissection**	1	1	0.701
**Ocular abnormalities**	14	2	0.009^a^
**Ectopia lentis**	14	2	0.009^a^
**Skeletal abnormalities**	23	14	0.832
**Long limbs**	22	14	0.605
**Thumb sign**	20	7	0.046^a^
**Wrist sign**	15	8	0.724
**Pectus deformities**	1	3	0.106
**Scoliosis**	0	1	0.372
**Hindfoot**	7	3	0.590
**Joint laxity**	1	1	0.109
**Facial abnormalities**	14	5	0.188
**Long and narrow head**	13	5	0.277
**High arched palate**	6	3	0.786
**Malar hypoplasia**	7	4	0.946
**Micro/retrognathia**	8	2	0.198

The Student *t* test (two-sided), chi-square, and Fischer’s exact test were used; *p* < 0.05 is considered significant

^a^Abnormalities are more common in FBN1 positive patients

Out of all cases (*n* = 131), 61 (46.56%) exhibited cardiovascular manifestations (Table 5). Table 4 illustrates the relationship between aortic root dilatation and other clinical features in children diagnosed with Marfan syndrome. There were no differences in the frequency of accompanying ocular, skeletal, and facial findings between the groups with and without aortic root dilatation.

**Table 5:** Relationship between aortic root dilatation and other clinical features in children diagnosed with Marfan syndrome

	***n*** **mean ± SD**	**Aortic root dilatation** **positive (*n* = 28)**	**Aortic root dilatation negative (*n* = 15)**	***p***
**Male/female**	32/11	21/7	11/4	0.904
**Age (year)**	12.56 ± 1.94	12.54 ± 5.43	10.8 ± 2.88	0.256
**Height SDS**	1.98 ± 0.56	1.60 ± 1.59	1.66 ± 1.46	0.908
***FBN1*** **(+)**	27	16	11	0.295
**Ectopia lentis**	16	9	7	0.347
**Skeletal abnormalities**	36	24	12	0.628
**Long limbs**	36	24	12	0.628
**Thumb sign**	27	16	11	0.295
**Wrist sign**	23	13	10	0.204
**Pectus deformities**	4	3	1	0.557
**Scoliosis**	1	0	1	0.547
**Hindfoot**	10	5	5	0.252
**Joint laxity**	2	1	1	0.538
**Facial abnormalities**	19	13	6	0.685
**Long and narrow head**	18	12	6	0.542
**High arched palate**	7	6	1	0.352
**Malar hypoplasia**	11	9	2	0.177
**Micor/retrognatia**	10	6	4	0.258

The Student *t* test (two-sided), chi-square, and Fischer’s exact test were used; *p* < 0.05 is significant

The distribution of *FBN1* variants based on three exon regions was as follows (Table 6). Table 6 displays the distribution of pathogenic variants and phenotypic abnormalities based on exon regions. The numbers of all variants were as follows: missense *n* = 29 (90.6%), frameshift *n* = 2 (6.4%), and nonsense *n* = 1 (3%). All cases with Cys-missense variants had aortic dilatation. The distribution of *FBN1* variant domains was as follows: EGF-like *n* = 15 (46.8%), cbEGF-like *n* = 9 (28%), and TGFb *n* = 8 (25.2%).

**Table 6:** Comparison of clinical manifestations based on exon regions in children with Marfan syndrome

	N-terminus Exon 1–21 (*n* = 9)	Middle region Exon 22–42 (*n* = 12)	C-Terminus Exon 43–65 (*n* = 7)	Non-coding region Intron 18, 47 (*n* = 4)	Total (*n*)
Age (year)	12.66 ± 5.02	11.41 ± 4.23	11.71 ± 7.67	12.25 ± 6.89	
Aortic root dilatation	6	5	3	2	16
Ectopia lentis	2	8	2	2	14
Long limb	2	8	**10**	5	25
Thumb/wrist sign	3	7	9	4	23
Long and narrow face	2	4	6	3	15
EGF-like	7	6	2	0	15
TGFβ	2	5	0	1	8
cbEGF-like	0	1	5	3	9
Variant type					
Missense	7	12	6	4	29
Frameshift	1	0	1	0	2
Nonsense	1	0	0	0	1

*cbEGF* calcium binding epidermal growth factor, *TGF* transforming growth factor

The frequency of the most common *FBN1* pathogenic variants was determined as follows: c.1786 T > C/p.Cys596Arg (exon 15) (*n* = 4, 12.5%) and c.7964C > T/p.Ala2655Val (exon 64) (*n* = 4, 12.5%), and c.4450A > G/p.N1484D (exon 36) (*n* = 2, 6.25%) (Table 7). Table 7 also shows the association of pathogenic variants with skeletal and facial abnormalities.

**Table 7:** Clinical phenotypic abnormalities and molecular findings in children with Marfan syndrome

								**Clinical manifeastations**												
**Protein change** **Nucleotidchangers**	**Age**	**Height SDS**	**n**	**Exon** **Intron**	**Domain**	**Variant type**	**Family history**	**CVS**				**Eye**	**Skeletal**			**Facial**			**ClinVar**	**Variant classificaiton based on ACMG**
								**AD**	**MVP**	**MR**	**TR**	**EL**	**LL**	**TWS**	**HD**	**LF**	**MH**	**MRG**		**Class 5, pathogenic** **(*n* = 26)**
p.Cys2040fs c.6119_6120del (rs1057517851)	5	2.16	1	49	Cterminal EGF-like	Frameshift (nullvariant)	** + **	**-**	** + **	** + **	** + **	**-**	** + **	** + **	** + **	** + **	** + **	**-**	Pathogenic	PVS1, PM2, PS3, PP3
p.Cys790Tyr c.2369G > A (rs193922188)	10	2.56	1	20	Cterminal -EGF-like	Missense	**-**	**-**	** + **	** + **	**-**	** + **	** + **	** + **	**-**	** + **	**-**	**-**	Pathogenic Likely pathogenic	PS1, PM1, PM2, PM5, PM6, PS3, PP3
p.Gly1013Arg c.3037G > A rs140593	15	1.73	1	25	TGFβ (TB)	Missense	**-**	** + **	** + **	** + **	**-**	** + **	** + **	** + **	**-**	**-**	**-**	**-**	Pathogenic Likely pathogenic VUS	PS1, PM2, PM5, PM6, PP3, PP4, PS3
p.Cys220VfsTer109 c.657_660delGGGG rs757505269	14	3.04	1	6	TGFβ	Frameshift (nullvariant)	**-**	** + **	** + **	** + **	**-**	**-**	** + **	** + **	**-**	** + **	**-**	**-**	Pathogenic	PVS1, PM2, PM6
p.Arg215Ter c.643C > T (rs111687884)	17	3.75	1	7	TGFβ	Missense	**-**	** + **	**-**	** + **	** + **	**-**	** + **	** + **	**-**	** + **	** + **	** + **	Pathogenic	PS1, PM2, PM4, PM6, PP3
p.Cys2295Arg c.6883 T > C (rs1555394644)	2	2.53	1	57	EGF-like	Missense	** + **	** + **	**-**	** + **	**-**	**-**	** + **	**-**	**-**	**-**	**-**	**-**	Pathogenic	PM1, PM2, PM5, PP2, PP3, PS3
p.Gly880Ser c.2638G > A (rs794728194)	11	2.18	1	22	TGFβ	Missense	**-**	**-**	** + **	**-**	**-**	** + **	** + **	** + **	**-**	** + **	**-**	** + **	Pathogenic Likely pathogenic	PM2, PM5, PM6, PP2, PP3, PS3
p.Cys596Arg c.1786 T > C	7 8 18 18	1.05 0.58 1.98 1.58	4	14	EGF-like2	Missense	**-**	**-** ** + ** ** + ** ** + **	** + ** ** + ** **-** ** + **	**-** ** + ** **-** **-**	**-** **-** **-** **-**	** + ** ** + ** ** + ** **-**	**-** **-** ** + ** **-**	**-** **-** ** + ** **-**	**-** **-** **-** **-**	**-** **-** ** + ** **-**	**-** **-** **-** **-**	**-** **-** **-** **-**	Pathogenic	PS1, PM1, PM2, PM5, PM6,P P3
p.Arg165Ter c.493 C > T rs113905529	11	1.94	1	5	EGF-like	Nonsense Nullvariant	**-**	**-**	** + **	**-**		** + **	** + **	** + **	**-**	**-**	**-**	**-**	Pathogenic Likely pathogenic	PVS1, PM2, PM4, PP3, PS3
p.Gly1127Val c.3380G > T rs1064794882	16	3.31	1	28	EGF-like	Missense	**-**	** + **	** + **	** + **	**-**	** + **	** + **	** + **	**-**	**-**	**-**	**-**	Likely pathogenic	PM1, PM2, PM5, PM6, PP2, PP3, PS3
p.Asn1484Asp c.4450 A > G rs76551543	11 4	133 3.79	2	36	EGF-like	Missense	**-**	**-** ** + **	**-** ** + **	**-** **-**	**-** **-**	**-** ** + **	**-** ** + **	** + ** **-**	**-** **-**	** + ** **-**	**-** **-**	**-** **-**	VUS	PS1, PM1, PM2, PM5, PM6, PP2, PP3, PS3_Supportive
p.Pro2175Leu c.6524 C > T	8	3.97	1	54	cbEGF-like	Missense	**-**	**-**	** + **	** + **	**-**	**-**	** + **	** + **	** + **	** + **	** + **	** + **	VUS	PS1, PM1, PM2, PM5, PM6, PP2, PP3, PS3_Moderate
p.Ala2655Val c.7964C > T rs202240409	13 7 8 18	3.24 1.77 1.58 0.98	4	64	cbEGF-like	Missense	**-**	** + ** ** + ** **-** **-**	** + ** **-** ** + ** ** + **	** + ** **-** ** + ** **-**	**-** **-** **-** **-**	** + ** **-** **-** ** + **	** + ** ** + ** ** + ** **-**	** + ** ** + ** ** + ** **-**	**-** **-** ** + ** **-**	** + ** **-** ** + ** **-**	** + ** ** + ** **-** **-**	** + ** ** + ** ** + ** **-**	Likely benign Benign VUS	PVS1, PS1, PM1, PM2, PM5, PP2, BS3
p.Pro1148Ala** c.3442 C > G 56 rs140598	13 9 13	0.34 2.43 2.86	3	28	EGF-like	Missense	**-**	** + ** **-** ** + **	** + ** ** + ** ** + **	** + ** ** + ** ** + **	** + ** **-** **-**	**-** **-** ** + **	**-** ** + ** ** + **	**-** ** + ** ** + **	**-** ** + ** **-**	**-** **-** ** + **	**-** **-** **-**	**-** **-** ** + **	Benign	PVS1, PS1, PM1, PM2, PP2, BS3
p.Thr1020Ala* c.3058 A > G rs111801777	18 3 17	2.52 1.77 1.74	3	25	TGFβ	Missense	** + **	**-** **-** **-**	**-** **-** **-**	**-** **-** ** + **	**-** **-** **-**	**-** **-** **-**	** + ** ** + ** ** + **	** + ** **-** ** + **	**-** **-** ** + **	** + ** **-** **-**	**-** **-** **-**	**-** **-** **-**	Likely benign VUS	PVS1, PS1, BP1, PP2, PM6, BP4
**Total (*n*)**							**3**	**13**	**18**	**15**	**3**	**12**	**20**	**18**	**5**	**12**	**5**	**7**		
																				**Class 4 (*n* = 1)**
p.Pro731Arg c.2192C > G rs961565152	10	2.79	1	15	EGF-like	Missense	**-**	**-**	**-**	**-**	**-**	**-**	** + **	** + **	**-**	**-**	**-**	**-**	Likely pathogenic	PVS1, PM1, PM2, PM5, PM6, PP2,PP3,PS3
**Total (*n*)**							**0**	**0**	**0**	**0**	**0**	**0**	**1**	**1**	**0**	**0**	**0**	**0**		
																				**Class 3 (*n* = 1)**
p.Glu810Val c.2429A > T	10	1.84	1	23	cbEGF-like	Missense	**-**	**-**	**-**	**-**	**-**	**-**	** + **	**-**	**-**	**-**	**-**	**-**	VUS	PS1, PM1, PM2, PM5, PM6, PP2, PP3
**Total (*n*)**							**0**	**0**	**0**	**0**	**0**	**0**	**1**	**0**	**0**	**0**	**0**	**0**		
**Intronic variants**				**Intron**																***N* = 4**
- c.2168-2A > G	5	3.87	1	18	TGFβ	Missense	+	-	+	+	+	+	+	+	-	+	+	+	Pathogenic	PS1, PM2, PP2
- c.5789-1G > A	1 18	1.47 1.98	2	47	cbEGF-like	Missense	-	+ +	- -	- +	+ +	- -	+ +	+ +	+	- +	- +	- -	Pathogenic Likely pathogenic	PS1, PM2, PM6, PP2
- c.5788 + 5 G > A (rs193922219)	14	2.54	1	47	cbEGF-like	Missense	-	+	+	+	+	+	+	+	+	+	+	+	Pathogenic Likely pathogenic	PS1, PM2, PM6, PP2, PP3, PS3
**Total (*n*)**			**4**				**1**	**3**	**2**	**3**	**4**	**2**	**4**	**4**	**2**	**3**	**3**	**2**		
**Total FBN1 positive group**			**32**				**4**	**16**	**20**	**18**	**7**	**14**	**26**	**23**	**7**	**15**	**8**	**9**		

*CVS* cardiovascular signs, *AD* aortic dilatation, *MVP* mitral valve prolapse, *MR* mitral valve regurtation, *TR* tricuspid valve regurtation, *EL* ectopia lentis, *TWS* thumb/wrist sign, *HD* heel deformity, *LF* long face, *MH*molar hypoplasia, *MRG* microganti/retrognaita, *EGF* epideraml growth factor, *cbEGF*calcium binding epidermal grawth factor, *TGFβ* transforming growth factor

^a^There are also cases reported as variant of uncertain significance (VUS) in the Clinvar database

^b^Reported as benign (Class 1) in all databases

## Discussion

This study evaluates the genotype-phenotype association in children with Marfan syndrome. There have been few published reports on this subject in children to date [22, 23]. It contributes to the limited number of pediatric reports that include genotype-phenotype associations in Marfan syndrome. This study revealed that the most common cardinal finding in children with MFS is cardiac anomalies. The Ghent criteria present diagnostic challenges in children, and there was no difference in the frequency of cardiac findings between *FBN1*-positive and *FBN1*-negative children. Ocular findings were more common in children with *FBN1*-positive MFS. Additionally, although the study did not have a large enough sample size to establish a well-defined genotype-phenotype relationship, it provides data for clinical phenotypes associated with some exonic regions/domains.

Previous studies have reported varying rates of *FBN1* variant positivity in cases screened with suspicion of MFS. In a study screening for suspected MFS, the *FBN1* variant was identified in 131 (40%) of 327 pediatric patients [11]. Out of the cases, 202 (61.17%) were diagnosed with Marfan syndrome (MFS) using the revised Ghent criteria. Among these cases, 39% had a clinical diagnosis. The average age at which genetic screening was conducted was 7.8 ± 5.4 years. In our study, an *FBN1* variant was identified in 32 out of 131 participants (24.4%). The detection rate of *FBN1* variants appears to be relatively low. Although the rate reported is acceptable compared to other studies (40% in Stark et al.), the data are not directly comparable [23]. The referenced study mainly reported (likely) pathogenic variants, while our study included all variants in the calculations. Additionally, the number of children diagnosed with the Ghent criteria was lower (35.5%) compared to the previous study. In another study conducted in adults at a different center, *FBN1* variant positivity (*n* = 51) was found to be 67.1% in 76 patients who underwent genetic screening [23]. This difference seems to be due to variances in patient selection. In our study, another reason for the low *FBN1* positivity rate may be that screening indications were broadened in children presenting with tall stature compared to their midparental height. Based on the data from the literature, it is clear that selecting the suitable cases for genetic screening is crucial. Out of the cases, 16 (37.2%) were diagnosed based on the Ghent criteria without detecting any *FBN1* variant. As mentioned above, low *FBN1* positivity has been reported in children diagnosed with MFS in previous studies. There could be several reasons for this low *FBN1* positivity. These patients may have one of the MFS-like fibrillinopathies, but unfortunately, they were not screened for these diseases. Another reason could be the diagnostic challenges posed by the Ghent criteria in children, or there may be differences in the definition of clinical phenotypes that underlie the Ghent criteria [11, 17, 24].

On the other hand, while an *FBN1* variant was detected in 5 cases, they were not diagnosed according to the Ghent criteria. This suggests that the presentation of MFS with characteristic clinical findings that develop with age, and the use of Ghent criteria in childhood, may lead to diagnostic inconsistencies. It is stated that the Ghent criteria are not reliable in children [24]. These findings should be followed up on, as specific changes will occur with age. This needs to be considered when evaluating the Ghent criteria in the childhood age group. Similarly, the age-dependent appearance of these findings also leads to differences in genotype-phenotype correlation. In a study involving the adult age group, the rate of patients diagnosed with MFS using the Ghent criteria was reported as 93.3% [16]. This high rate could be due to the adult age of the subjects, with a mean age of 41.33 ± 3.77. Therefore, cross-sectional studies in childhood may not fully reveal the genotype-phenotype relationship [24].

Previous studies have reported an association between the severity of cardiac findings and frameshift and nonsense variant types [25, 26]. However, in our study, we did not find any association with cardiac or other clinical manifestations. This lack of difference may be due to the relatively small number of cases in our cohort. Additionally, the age of the cases may also contribute to this discrepancy. The presence of cardiac findings and their impact on life expectancy, as well as the rate of progression of aortic dilatation, may vary with advancing age.

The variant p.Pro1148Ala c.3442 C > G was reported as benign in the ClinVar database. Cardiac findings were detected in all 3 cases with this variant, and EL was also present in one case. In silico analysis also classified it as benign strong. This variant is a frequently observed polymorphism in previous studies [27]. In our study, these 3 cases were diagnosed as MFS cases according to the Ghent criteria. Unfortunately, parental segregation analysis could not be performed for the same variant due to a possible presence of another variant in the case. Comprehensive bioinformatic re-analyses were planned for these 3 cases with the delegation, but the parents did not cooperate. Additionally, regulations of social security institutions posed an obstacle to this. Reanalysis of exon data should be standard practice for patients. For these cases, the reverse phenotyping approach may provide a potential breakthrough [28]. Today, reverse phenotyping appears to be a useful approach that will change our conventional understanding of establishing the genotype-phenotype relationship. While previous approaches have focused on assigning phenotypes to individuals with genetic disorders, the reverse phenotyping approach involves recruiting individuals with a certain genotype and analyzing hypotheses for possible predicted phenotypes. Recognizing phenotypes from genotypes offers a rational tool for comprehensive insight into the molecular classification of diseases.

When evaluating the concordance between genotype and phenotype based on pathogenicity classes according to ACMG, although a well-established phenotypic association with some exon regions and pathogenic variants is known in patients with MFS, there are also incompatibilities in some cases [2]. In the current study, the number of patients who participated is insufficient to definitively state that there is no well-established correlation between clinical findings and genotypes based on pathogenicity classes and exon regions in children with Marfan syndrome. Further research with a larger sample size is necessary to establish a strong genotype-phenotype relationship in children with MFS.

### Limitations

There are several limitations to this study. A comprehensive molecular panel analysis was not conducted to determine if patients diagnosed with MFS clinically had genetic variants associated with similar disorders such as Loeys-Dietz syndrome or isolated ectopia lentis. Only *FBN1* sequencing analysis was performed, making it impossible to distinguish between conditions like isolated ectopia lentis or MFS. The fact that neither a panel nor CNV-sequencing (or software equivalent, e.g., ExomeDepth) was used is a significant limitation, since patients with exon deletions or Loeys-Dietz syndrome could have been included in the *FBN1* negative group. This is especially important in the patient who presented with an aortic dissection and in the two patients with aortic dilatation and EL, in whom a pathogenic variant was not found. Additionally, the study was unable to test the tissue-specific expression of *FBN1* with different transcripts, which could be a potential cause of phenotypic variability [29]. Another limitation is the inability to perform CNV sequencing or other techniques to detect larger insertions/deletions in the *FBN1* gene. Similarly, the retrospective nature of the study constituted another limitation. Parental segregation analyses could not be performed when necessary. Of the patients with a family history, only 3 parents had previous *FBN1* analysis results, and they had the same pathogenic variant as the children with MFS. Unfortunately, pathogenic *FBN1* variants detected with NGS could not be validated by Sanger sequencing in this genotype-phenotype incompatibility. Ultimately, this is a retrospective study, and genotype-phenotype discrepancies may result from differences in study designs, ranging from patient selection criteria to the definition of clinical phenotypes.

## Conclusion

This study presents data on diagnostic clinical findings of childhood MFS and associated FBN1 variants. It indicates that the Ghent diagnostic criteria, which include clinical findings and FBN1 variant features, have some diagnostic difficulties in childhood MFS. It also emphasizes the importance of performing bioinformatic reanalysis to evaluate the genotype-phenotype relationship due to the changing nature of clinical phenotypes with age.

## Data Availability

The datasets generated during and/or analyzed during the current study are available from the corresponding author on reasonable request.
